# Tumor-associated macrophages remodel the suppressive tumor immune microenvironment and targeted therapy for immunotherapy

**DOI:** 10.1186/s13046-025-03377-9

**Published:** 2025-05-16

**Authors:** Yan Yang, Sijia Li, Kenneth K.W. To, Shuangli Zhu, Fang Wang, Liwu Fu

**Affiliations:** 1 State Key Laboratory of Oncology in South China, Guangdong Provincial Clinical Research Center for Cancer, Sun Yat-sen University Cancer Center, Guangzhou, 510060 P. R. China; 2 School of Pharmacy, The Chinese University of Hong Kong, Hong Kong, 999077 P.R. China

**Keywords:** Tumor-associated macrophages, Tumor microenvironment, Immune evasion, Immunotherapy, Immune checkpoint inhibitors

## Abstract

Despite the significant advances in the development of immune checkpoint inhibitors (ICI), primary and acquired ICI resistance remains the primary impediment to effective cancer immunotherapy. Residing in the tumor microenvironment (TME), tumor-associated macrophages (TAMs) play a pivotal role in tumor progression by regulating diverse signaling pathways. Notably, accumulating evidence has confirmed that TAMs interplay with various cellular components within the TME directly or indirectly to maintain the dynamic balance of the M1/M2 ratio and shape an immunosuppressive TME, consequently conferring immune evasion and immunotherapy tolerance. Detailed investigation of the communication network around TAMs could provide potential molecular targets and optimize ICI therapies. In this review, we systematically summarize the latest advances in understanding the origin and functional plasticity of TAMs, with a focus on the key signaling pathways driving macrophage polarization and the diverse stimuli that regulate this dynamic process. Moreover, we elaborate on the intricate interplay between TAMs and other cellular constituents within the TME, that is driving tumor initiation, progression and immune evasion, exploring novel targets for cancer immunotherapy. We further discuss current challenges and future research directions, emphasizing the need to decode TAM-TME interactions and translate preclinical findings into clinical breakthroughs. In conclusion, while TAM-targeted therapies hold significant promise for enhancing immunotherapy outcomes, addressing key challenges—such as TAM heterogeneity, context-dependent plasticity, and therapeutic resistance—remains critical to achieving optimal clinical efficacy.

## Highlights

Provide a comprehensive understanding of TAM origin and biomarkers of M1 and M2.

Chemokines and interleukins play key roles in macrophage polarization.

TAM-TME interactions are responsible for tumor progression and immune evasion.

TAM-targeted therapies are promising therapies with heterogeneity and plasticity.

## Introduction

The landscape of cancer immunotherapy has evolved from broad immune activation—exemplified by vaccines and cytokine therapies—toward precise immune normalization which is designed to restore intrinsic antitumor immunity [1]. In contrast to broad immune activation, immune normalization focuses on targeting tumor-induced immune escape mechanisms and regulating immune cell balance between pro-tumor and anti-tumor immune cells [1, 2]. Immune checkpoint inhibitors (ICIs), a cornerstone of immune normalization, primarily act by blocking immune checkpoints (e.g., the PD-1/PD-L1 pathway) to reactivate CD8 + T cell-mediated antitumor immunity. To date, several ICIs have been developed and they gave rise to remarkable clinical outcomes across malignancies, offering superior efficacy and reduced adverse events compared to immune enhancement [3–7]. However, ICIs are still severely limited due to primary and acquired resistance [8, 9]. A multifaceted array of factors, including driver oncogene mutations, epigenetic alterations, disruptions in critical signaling pathways, defects in the antigen-presenting pathway and immune evasion, are known to contribute to diminished effectiveness of immunotherapy [10–12]. To improve the efficacy of ICIs, there is an urgent need to unveil the mechanisms of drug resistance and explore novel molecular targets.

The term tumor microenvironment (TME) was first coined by Paget in 1889 to describe the dynamic ecosystem harboring tumor cells within a diverse cellular landscape, which is tightly related to the responses to anti-tumor drugs and therapeutic resistance [13]. The TME incorporates diverse immune cells and fibroblasts, collectively embedded in a modified, vascularized extracellular matrix [14], among which tumor-associated macrophages (TAMs) have garnered substantial attention owing to their significant involvement in carcinogenesis, tumor advancement and immune escape [15].

Generally, TAMs undergo the process known as “macrophage polarization” and polarize into M1 or M2 macrophages. M2-like TAMs have been shown to foster an immunosuppressive TME by secreting soluble factors (e.g., cytokines, exosomes) and expressing inhibitory surface proteins, subsequently leading to ICI resistance [16, 17]. These properties position TAMs as promising therapeutic targets for immune normalization and overcoming resistance to ICIs. While numerous investigations have disclosed the link between TAMs and immune evasion, the specific mechanisms of interactions between TAMs and diverse reshaped TME components remain elusive.

This review aims to systematically describe the origin, heterogeneity and functional plasticity of TAMs, summarize the possible molecular mechanisms of TAM-mediated immune evasion based on interactions among TAMs and other cells, and highlight emerging therapeutic strategies to target TAMs for synergizing with ICIs. We further discuss current challenges and future directions in targeting TAMs to reverse immunosuppression and optimize clinical outcomes.

### Origin and recruitment of TAMs

Macrophages are present in nearly all tissues, primarily derived from circulating monocytes differentiated from hematopoietic stem cells, and partially stem from yolk sac and fetal liver progenitors [18]. Hematopoietic stem cells (HSC) initially transform into bone marrow progenitor cells, subsequently undergo the granulocyte/macrophage-restricted progenitor cells stage, and further differentiate into macrophage/dendritic cell progenitors, ultimately equipped with the potential to differentiate into monocytes and mononuclear myeloid suppressor cells (M-MDSC) [19]. Instead, embryonic tissue-resident macrophages are shown to proliferate and differentiate in situ throughout their life. Upon activation of inflammatory signals, myelopoiesis is induced in the bone marrow, prompting the release of monocytic precursors into the bloodstream, which would ultimately reach tissues and organs, and either mature into bone marrow-derived macrophages or remain in an immature state, designated as MDSCs [20]. A recent study has proven that bone marrow-derived macrophages coexist with tissue-resident macrophages proliferating in situ in the brain, lung and spleen, which explains the diverse origins and functions of these cells [21].

Serving as the vanguard of the immune system, macrophages perform a diverse array of functions in the preservation of tissue homeostasis, the neutralization of pathogenic threats, and the modulation of inflammatory responses [22]. Specialized in phagocytosis, these cells safeguard the organism by engulfing and digesting invading pathogens, cellular debris, and other foreign substances, thereby constituting the fundamental component of nonspecific immunity (innate immunity) [23]. Concurrently, they release cytokines and chemokines to regulate immune responses and facilitate the initiation of specific defense mechanisms by processing and presenting antigens (adaptive immunity), thereby acting as a bridge between innate and adaptive immunity [24].

**Fig. 1:**
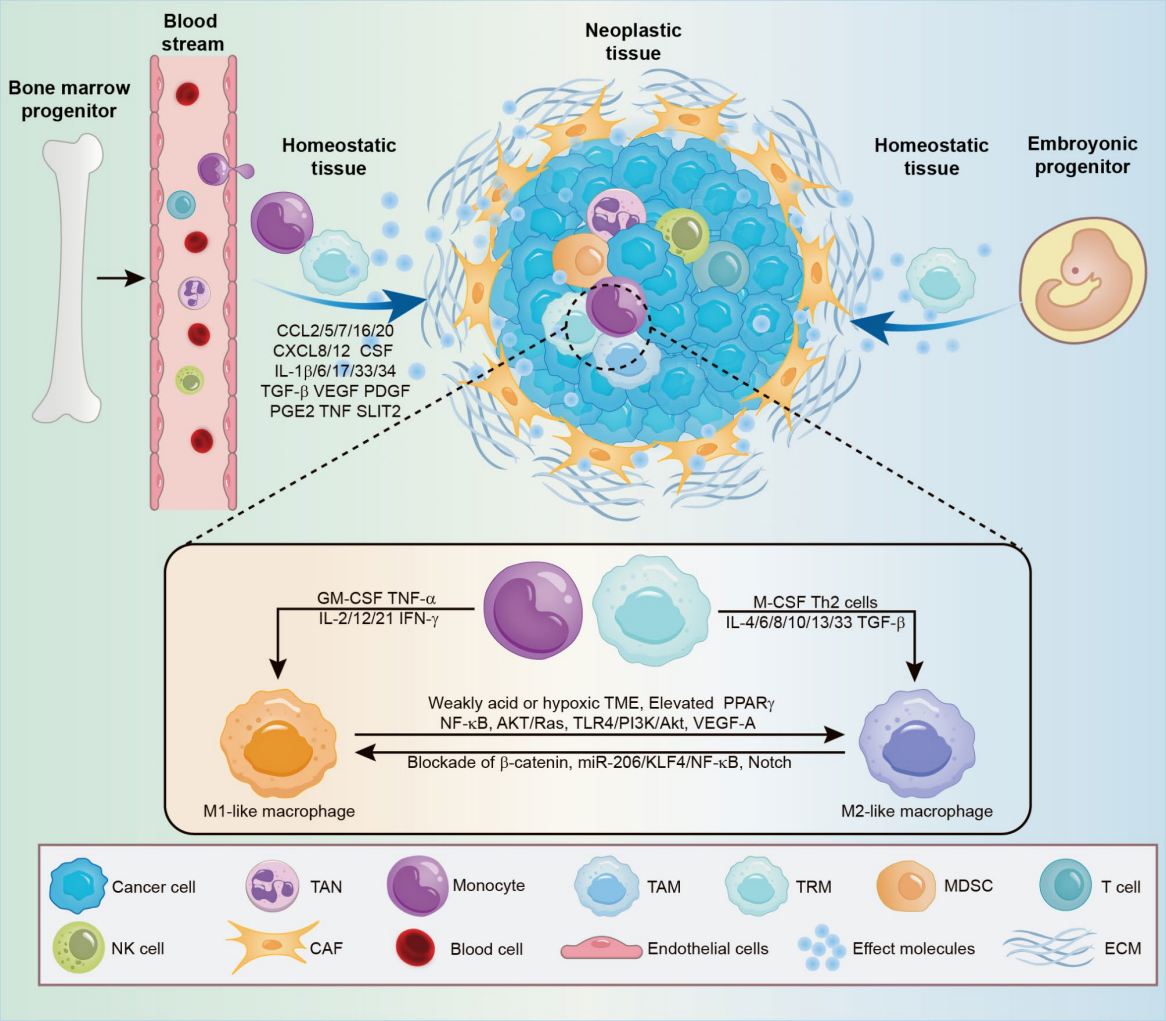
The origin and evolution of TAMs. TAMs stem from bone marrow-derived circulating monocytes and embryonic tissue-resident macrophages (TRM). Regulatory molecules (including CCL2, CCL5, CCL7, CCL16, CCL20, CXCL8, CXCL12, CSF, IL-1β, IL-6, IL-17, IL-33, IL-34, TGF-β, TNF, PGE2, VEGF, PDGF and SLIT2) induce the recruitment of monocytes/macrophages to tumor tissues. Upon recruitment to the TME, these cells are stimulated by various signals to polarize into M1 or M2-like macrophages and then exhibit diverse functions

TAMs represent a major component of the TME, which are macrophages activated in the surrounding of tumor cells [25]. During the initial phase of neoplastic transformation, proinflammatory immune cells, including monocytes, are recruited by early inflammatory signals. Additionally, these signals may also activate embryogenic tissue-resident macrophages present within the local microenvironment [20]. (Figure. 1)

It is widely acknowledged that chemokines secreted by tumor cells such as CCL2, CCL5, CCL7, CCL20, CXCL2, CXCL8 and CXCL12 [15, 26–28] significantly contribute to the recruitment of monocytes and macrophages during neoplastic transformation, among which CCL2 plays a key role in TAM recruitment. In fact, the secretion of CCL2 by tumor cells has been observed in a variety of cancer types, such as esophageal carcinoma [29], bladder cancer [30], breast cancer [31], melanoma [32], and colorectal cancer (CRC) [33], leading to enhanced TAM infiltration in TME. On the other hand, CCL2-induced macrophages are known to produce VEGF-A and activate the AKT signaling pathway to increase STC1 expression in melanoma, thereby contributing to YAP activation and subsequent CCL2 upregulation [32]. Moreover, other signaling molecules such as CSF-1, CSF-2, IL-6, IL-17, IL-33, IL-34, transforming growth factor-beta (TGF-β), vascular endothelial growth factor (VEGF), and SLIT2 also facilitate the infiltration and polarization of TAMs [34–40]. Recent reports also unveil the role of PI3K-AKT-SELE/VCAM1 axis and CCL16-CCR1 axis in TAM recruitment in pancreatic ductal adenocarcinoma (PDAC) and hepatocellular carcinoma (HCC) respectively [41, 42]. Interestingly, TAMs are reported to maintain effective crosstalk with other cells to reinforce their recruitment to the TME. For example, pancreatic cancer cells secrete PGE2 and TNF to enhance macrophage recruitment to the TME. On the other hand, macrophages infiltrating the TME are known to induce inflammatory responses of adjacent PDACs, which promotes further PGE2 and TNF production via IL-1β signaling and recruits more TAMs [43]. This may vividly show how TAMs interact with tumor cells to form a vicious circle.

Apart from tumor cells, other cell types are also involved in TAM recruitment. Zhou et al. recently reported that various chemokines (including CCL2, CCL5, and CSF1) are secreted from tumor-associated neutrophils (TANs) to orchestrate TAM infiltration. Meanwhile, TAMs also secrete some TAN-chemo-attractants such as CXCL8 and CSF3. Notably, some of these chemokines, such as CSF1 and CXCL8, would further increase upon the co-culturing of TANs and TAMs. There may be a reciprocal stimulatory relationship between TANs and TAMs, which could explain their spatial association [44]. Furthermore, cancer-associated fibroblasts are shown to enhance TAM recruitment via the CXCL12/CXCR4 axis in breast cancer [45] and the IL-8/CXCR2 axis in CRC [46].

### Biological characteristics and functions of TAMs

In response to specific stimuli and signals, naïve macrophages (M0) are known to adopt distinctive functional phenotypes via macrophage polarization. In general, M0 macrophages can be polarized into two subtypes, namely classically activated M1 macrophages which are characterized by the release of proinflammatory factors, and alternatively activated M2 macrophages associated with anti-inflammatory cytokines [47]. The specific differences are shown in Table 1 [15, 29, 48–54]. Upon their entry into the TME, monocytes or tissue-resident macrophages will differentiate into either pro-tumor or anti-tumor macrophages with the help of regulatory molecules from tumor cells. (Table 2) [55–85]. In general, interleukins like IL-2, IL-12, IL-21 facilitate M1 polarization [86], while M0 macrophages polarize into M2-like phenotype via the stimulation of cytokines and chemokines such as CCL2 and VEGF in HCC [87, 88], IL-4/13 in various cancers, IL-6 in HCC [89], breast cancer [55], CRC [90] and GBM [91], IL-8 in pancreatic cancer [82], lung cancer [92] and esophageal squamous cell carcinoma [93], IL-10 in CRC [79] and gastric cancer [94], IL-33 in esophageal squamous cell carcinoma [95], gastric cancer [96], lung cancer [97] and CRC [98], IL-34 in breast cancer [99] and HCC [100]. M1-like macrophages exert proinflammatory functions and activate T cells to defend against pathogens via the secretion of inflammatory factors [101]. Therefore, these cells play a vital role in the initiation of immune responses in most types of tumors. However, emerging evidence underscores that M1-like macrophages may also induce inflammatory TME and promote tumor initiation and progression via the secretion of inflammatory factors, such as IL-1β in CRC [102] and pancreatic cancer [43]. In addition, accumulating evidence reveals that M1 macrophages contribute to cancer stemness and immune evasion in oral squamous cell carcinoma [103], glioma [104], breast cancer [105], HCC [106]. On the other hand, M2-like macrophages secrete anti-inflammatory factors to maintain tissue homeostasis. These cells are known to suppress the immune responses against tumors through diverse mechanisms. For example, M2 macrophages are shown to lead to the dysfunction of cytotoxic cells like CD8 + T cells and NK cells and attract regulatory T cells to promote tumor progression [73, 107, 108]. Surprisingly, these cells may also be beneficial to cancer therapy due to their anti-inflammatory functions and ability of tissue repairing [109, 110]. These emphasize the heterogeneity and complexity of macrophages.

**Table 1:** Phenotypes and biomarkers of macrophages

Type	Stimuli	Biomarker	Overall function(s)
M1	LPS, GM-CSF, TNF-α, IFN-γ	MHC ΙΙ^hi^, CD80, CD86, iNOS, HLA-DR, SOCS3, TLR2, TLR4, IL-1R, FCGR1A	a. Produce pro-inflammatory cytokines b. Occupy a key role in Th1 cell recruitment, pathogen resistance, and tumor-killing
M2	M-CSF, IL-4, IL-13, IL-10, TGF-β	CD163, CD206, CD204, CD209, CD115, CD301, Arg1, MACRO, TLR1, TLR7, TLR8	a. Suppress inflammation b. Induce a Th2 response and improve tumor progression

Abbreviations: LPS = lipopolysaccharide; GM-CSF = granulocyte-macrophage colony-stimulating factor; TNF-α = tumor necrosis factor-α; IFN-γ = interferon-γ; M-CSF = macrophage-colony stimulating factor

**Table 2:** TAMs differentiation under the influence of tumor microenvironment

Tumor type	Pathway	Differentiation	Biological function(s)	Ref
BC	MCT-1/miR-34a/IL-6/IL-6R	Pro-M2 polarization	Promote EMT and stemness	[55]
	MiR-138-5p /KDM6B	Anti-M1 + Pro-M2 polarization	Promote metastasis to the lung	[56]
	Lactate/Gpr132	Pro-M2 polarization	Facilitate tumor adhesion, migration, and invasion	[57]
	Lactate/HIF-1α/STAT3	Pro-M2 polarization	Lead to endocrine therapy resistance	[58]
	MiR-200c/PAI-2 q15.6	Pro-M2 polarization	Promote the cell migration ability	[59]
	PS/MERTK/STAT3/JMJD3/IRF4	Pro-M2 polarization	Promote tumor growth	[60]
Ovarian cancer	CircITGB6/IGF2BP2/FGF9	Pro-M2 polarization	Mediate resistance to cisplatin	[61]
	CircATP2B4/miR-532-3p/SREBF1/PI3Kα/AKT	Pro-M2 polarization	Promote tumor metastasis	[62]
	ETS1/αvβ5/AKT/Sp1	Pro-M2 polarization	Promote omental metastasis	[63]
LCA	CSF1R/AKT	Pro-M2 polarization	Suppress antitumor immune responses	[64]
	Src/CD155/MIF	Pro-M2 polarization	Facilitate distant metastasis	[65]
	Succinate/SUCNR1/PI3K-HIF-1α	Pro-M2 polarization	Promote tumor metastasis	[66]
	PIM1/NF-κB/CCL2/CCR22	Pro-M2 polarization	Promote tumor progression and immune evasion	[67]
	MiR-19b-3p/PTPRD/STAT3	Pro-M2 polarization	Promote tumor metastasis	[68]
	MiR-21-5p/PTEN	Anti-M2 polarization	Attenuate apoptotic and promote metastasis	[69]
GC	TLR4/PI3K/Akt	Pro-M2 polarization	Increase the migration and invasion abilities	[70]
	PLXNC1/MEK1/MSK1/CREB1/miR-92b-5p/STAT3	Pro-M2 polarization	Promote tumor growth	[71]
	CircATP8A1/miR-1-3p/STAT6	Pro-M2 polarization	Promote proliferation and migration	[72]
HCC	AKT/Ras	Pro-M2 polarization	Impair enrichment of cytotoxic T cells	[73]
	MiR-206/KLF4/NF-κB	Pro-M1 polarization	Enhance expansion and migration of CD8 + T cells	[73]
CRC	PKN2/DUSP6-Erk1/2	Anti-M2 polarization	Inhibit tumor growth	[74]
	SPON2/integrin β1/PYK2	Pro-M2 polarization	Promote tumor growth and metastasis	[75]
	IL10R/Wnt5a/CaMKII/ERK/STAT3/IL-10	Pro-M2 polarization	Promote tumor growth and metastasis	[76]
	CTSK/TLR4/PI3K/AKT/mTOR	Pro-M2 polarization	Promote migration and motility ability of CRC cells	[77]
	HnRNPA1miR-106a-5pJAK2/STAT3	Pro-M2 polarization	Promote CRC liver metastasis	[78]
	circPOLQ/miR-379-3p/IL-10/STAT3	Pro-M2 polarization	Promote tumor invasion and migration	[79]
PC	FGD5-AS1/p300/STAT3/NF-κB	Pro-M2 polarization	Promote tumor progression	[80]
	Lactate/Gpr132	Pro-M2 polarization	Support growth and metastasis	[81]
	ANXA2/MMP28/MAPK/JNK/IL-8	Pro-M2 polarization	Promote tumor growth and migration	[82]
Melanoma	IL-1R-MYD88-Tet2	Pro-M2 polarization	Attract MDSCs	[83]
NPC	USP7/TRIM24/SPLUNC1	Pro-M1 polarization	Repress tumor growth and migration	[84]
HNSCC	miR-9/PPARδ/NF-κB	Pro-M1 polarization	Increase the radiosensitivity of HPV + HNSCC	[85]
GBM	SLIT/ROBO/PI3K-γ	Pro-M2 polarization	Facilitate glioma growth and vascular dysmorphia	[38]

Abbreviations: PS, phosphatidylserine; CTSK, cathepsin K; EMT, epithelial-mesenchymal transition; BC, breast cancer; LCA, lung cancer; GC, gastric cancer; HCC, hepatocellular carcinoma; CRC, colorectal cancer; PC, pancreatic cancer; NPC, nasopharyngeal carcinoma; HNSCC, head and neck squamous cell carcinoma; GBM, glioblastoma

### Plasticity of TAM polarization

TAMs in TME are in a constant state of transition under the regulation of different signaling pathways. Several cellular signaling pathways are also involved in the polarization of M0 macrophages. Macrophages are known to interact with stimuli from TME and transfer them to nuclear compartments through membrane receptors and relevant signaling pathways to regulate the reprograming-related gene expression [111]. For example, activation of TLR4/5/NF-κB induced by pathogen-associated molecular patterns (PAMPs) ligands including LPS are shown to promote an M1-like phenotype and defend against tumors [112, 113]. Pim-1 proto-oncogene (PIM1), p50, bone marrow differentiation factor 88 (MyD88) and interferon regulatory factor 5 (IRF5) are engaged in the stimulation of NF-κB protein, which would translocate to the nuclear compartments and promote the transcription of proinflammatory genes [67, 114, 115]. In addition, the binding of IFN-γ with its membrane receptors stimulates JAK/STAT pathway downstream, promoting the phosphorylation of STAT1 and its translocation to the cell nucleus [116]. STAT1 is positively regulated by tumor necrosis factor superfamily-15 (TNFSF15) [117], tripartite motif-containing protein 65 (TRIM65) [118], integrin beta3 signaling [119] and negatively by the suppressor of cytokine signaling (SOCS) [120]. Similarly, it has been proven that oxidative stress caused by alanine-serine-cysteine transporter 2 (ASCT2) deletion leads to the unregulated release of exosomal THBS1 from oral squamous cell carcinoma cells [121], which activates p38, Akt, and SAPK/JNK signaling and favor M1-like differentiation [122]. In terms of M2-like macrophages, both PI3K/Akt/mTOR and TGF-β/Smad signaling activated by TGF-β serve as important regulating mechanisms. TGF-β binds to its receptor and leads to the phosphorylation of Smad2/3, which translocates to the cell nucleus to regulate gene expression [111]. Akt is activated by PI3K-induced PIP3, subsequently activating mTOR in the cytoplasm. This pathway is negatively regulated by Interferon-stimulated exonuclease gene 20 (ISG20) [123]. Surprisingly, *CTSK*/TLR4 signaling are also shown to promote M2 polarization in an mTOC-dependent manner under the positive regulation of *SOAT1* [77, 124]. Another important regulatory pathway is the JAK/STAT pathway induced by IL-4 and IL-6, which triggers the activation of STAT3 and STAT6 and M2-like transformation. The inhibition of METTL3, HIF, FABP2/5, phosphatidylserine are proven to favor M2-like phenotype via STAT-dependent manner [60, 125–127]. Furthermore, the Notch pathway and Wnt/β-catenin signaling pathway also participate in the process [76, 111]. The pathways mentioned above work synergistically to favor M2-like features and exert pro-oncogenic functions.

Post-transcriptional regulation through non-coding RNAs (ncRNAs) also plays a vital role in TAM polarization. At the post-transcriptional level, exosomal non-coding RNAs (ncRNAs) like miRNAs, circRNAs and lncRNAs can also regulate TAM polarization by regulating key pathways and transcription factors [63]. For example, miR-9 and miR-30d-5p are able to trigger the activation of NF-κB signaling and drive M1 polarization via the inhibition of PPARδ and SOCS1 respectively [85, 128]. Similarly, exosomal miR-221 is also known to suppress SOCS1 to drive M1 transformation via the activation of the JAK/STAT pathway [129]. Targeting these miRNAs serves as a good choice to attenuate the inflammatory response and tumor progression.

On the other hand, certain ncRNAs have been identified to promote M2 polarization targeting JAK/STAT signaling pathways through the activation of STAT3, such as miRNAs like miR-92b-5p [71], miR-106a-5p [78], miR-19b-3p [68], circ-RNAs like circ-00142 [130], circ3-POLQ [79] and circ-ATP8A1 [72], lncRNAs like HAGLROS [131], FGD5-AS1 [80], and LINC00958 [132]. The inhibition SOCS7 and SOCS6 play a vital role in the activation of STAT3 and the subsequent transformation towards M2 macrophages [71, 78]. Other miRNAs, such as miR-361-3p in breast cancer [133], miR-934, miR-25-3p, miR-130b-3p, miR-425-5p in liver metastasis of CRC [134, 135] and miR-301a-3p in pancreatic cancer [136] are involved in TAM polarization through other mechanisms. These miRNAs are shown to target the PI3K/Akt signaling and inhibit the tumor suppressor protein PTEN, thereby increasing the expression of M2-related genes such as VEGF [137–139].

Given the significant involvement of ncRNAs in the regulation of tumor progression and immune evasion and their promising clinical applications in various types of tumors, it is of great value to explore their functions and underlying mechanisms in depth. This will definitely enrich the understanding of their biological functions and provide ideal and personalized targeted strategies to defend against tumors.

Epigenetic modifications including DNA methylation and histone modifications are also associated with macrophage polarization. DNA methylation relies on DNA methyltransferases (DNMTs) to add a methyl group to a specific DNA position, thereby silencing the target gene. TAM infiltration correlates with DNMT1 expression in tumor tissues. Emerging evidence underscores the pivotal role of DNMTs in the modulation of macrophage phenotypes. For example, in breast cancer, elevated expression of DNMT1 is observed in M2 macrophages via the IL-6-pSTAT3-ZEB1-DNMT1 axis [140]. Enhanced DNMT1 may contribute to the hypermethylation of the TP53 promoter and inhibit p53 expression, which is an indicator of poor prognosis [141]. Likewise, acetylation of histones or transcription factors acts dynamically in the modulation of TAM phenotypes [130]. According to the research in lung cancer done by Yi-Chang Wang and colleagues, enhanced histone-3 acetylation and decreased levels of DNMT1 and IκB is mediated by USP24 via the stabilization of p300 and β-TrCP, thereby leading to elevated IL-6 transcription in M2 macrophages [142]. Histone deacetylases (HDACs) are also tightly linked to TAM phenotypes. It is known that HDAC3, HDAC6, HDAC7 and HDAC9 [143, 144] are identified to favor M1 macrophages while HDAC2, HDAC8, SIRT1, SIRT2, and SIRT6 are to facilitate M2 transformation [145–148].

Recently, cancer metabolism has received considerable attention due to its influence on tumor progression and immune regulation. Hypoxia and low PH have been observed in the microenvironment in various types of cancers, which are found to regulate TAM polarization. Generally, hypoxia-mediated elevated expression of HIF1α upregulates the key glycolysis-related enzymes like HK2, PFKP, PKM2 and G6PD to promote aerobic glycolysis and release more lactate in a MCT1/4-dependent manner [149–151]. Lactate receptors G protein-coupled receptor 81 (gpr81) and gpr132 on TAMs are able to sense the increased lactate production and drive pro-tumorigenic M2 polarization [81, 152, 153]. Interestingly, lactate is also involved in epigenetic modifications through the intervention of lactylation or other manners. For example, after being transported into TAMs, lactate evokes lactylation of the histone H3K18la site and stimulates pro-tumor macrophage activity [154]. Therefore, M1 polarization can be promoted by reducing the levels of lactate and protein lactylation [155]. In addition, HCC-derived 27-hydroxycholesterol 27HC and TNBC-derived glutaminase 1 enhance the level of lipid metabolism and glutamine metabolism in TAMs, respectively, thereby triggering M2 macrophage polarization and tumor invasion and progression [150, 156].

Notably, the majority of TAMs adopt an M2-like phenotype in most cancer types, which promotes tumor growth and suppresses anti-tumor immune response [19]. It is noteworthy that the polarization states are not immutable but plastic. There are many factors facilitating the differentiation of M1 macrophages towards M2, such as stimulation of the NF-κB signaling pathway, a weakly acid or hypoxic TME and elevated expression of proliferator receptor gamma (PPARγ), making it a promising strategy for cancer treatment [157]. A recent report verifies that inhibition of β-catenin promotes the transformation of M2-like TAMs towards M1-like TAMs [158]. Conversely, activated AKT/Ras signaling facilitates M1 to M2 polarization of Kuffer cells [73]. However, with technical progress, TAMs with dual characteristics of M1 and M2 have been identified, indicating the binary classification may be oversimplified. For example, Nicoletta et al. performed single-cell RNA-sequencing (scRNA-seq) on PDAC biopsies from cancer patients and revealed that inflammatory IL-1β + TAMs were shown to co-express both inflammatory (MHCII, CD80 and CD86) and immune inhibitory markers (CD206, arg-1 and PD-L1) [43]. To tackle the heterogeneity and complexity of TAMs in diverse tumor types and different tumor development stages, more TAM classification methods are needed to help with the understanding of the dynamic and intricate functions of TAMs, such as subtypes based on gene expression profiles [159, 160] or the dimension of time [161].

### Role of TAMs in tumor initiation and progression

Cancer-related inflammation is considered the seventh hallmark of cancer and promotes tumorigenesis. The pivotal role of TAMs in the link between inflammation and tumor development via the secretion of numerous cytokines/chemokines has been extensively investigated in preclinical and clinical studies [162, 163]. For example, TAM-derived IL-6, TNF-α and STAT3 signaling were believed to aggravate inflammation of TME and accelerate tumor advancement in PDAC and HCC [164–167]. M1-like macrophages typically exert anti-tumor effects by secreting proinflammatory factors such as IL-6/TNF-α in CRC [168, 169] and prostate cancer (PCA) [170], IL-1α/IL-6 in lung cancer [171]. In contrast, M2-like macrophages dominate the TME and drive immunosuppression through distinct mediators, such as IL-1β in CRC [168], IL-1β/IL-6/TNF-α in breast cancer [172]. This functional dichotomy highlights how the same cytokines (e.g., IL-6, TNF-α) may exhibit opposing roles depending on macrophage polarization states and TME context. In addition, a recent research pointed out that the inflammatory loop between PDACs and IL-1β-expressing TAMs with mixed M1 and M2 characteristics become a novel strategy for reprogram of inflammation and immune suppression [43], verifying the oversimplified of binary classification again.

Accumulating research shows that TAMs are engaged in tumor progression through diverse mechanisms [173]. TAMs are known to directly support tumor proliferation by expressing molecules including epidermal growth factor (EGF), TGF-β, and members of the fibroblast growth factor (FGF) family. Moreover, TAMs also secrete other factors such as VEGF, COX-2, and platelet-derived growth factor (PDGF), MMP2, MMP9 to augment blood vessel formation [174–176]. TAMs also participate in stromal remodeling, tumor invasion, and metastasis by producing several enzymes to degrade extracellular matrix (ECM), including several metalloproteinases (such as MMP-2, MMP-7, MMP-9, and MMP-12) as well as urokinase-type plasminogen activator. The deregulation of ECM facilitates proteolytic cleavages, enabling tumor cells to escape and disseminate, ultimately leading to metastasis [177, 178]. Furthermore, Hu et al. recently reported the role of IL6-STAT3-C/EBPβ-IL6 positive feedback loop in TAMs for facilitating the epithelial-mesenchymal transition (EMT) pathway, a process that significantly contributes to tumor metastasis [179]. Details are listed in Table 3 [32, 73, 76, 80, 102, 103, 164–167, 169, 179–194].

**Table 3:** The functions of TAMs in tumor initiation and development

Cell type	Tumor type	Secretion	Biological function(s)	Ref	
M1-like macrophages	HCC	CCL2	Enhance the CD8 + T cell recruitment	[73]	
	Melanoma	CCL20, CXCL8-11, IL-1b, IL-6, TNF-a	Promote inflammatory response, apoptosis, and cell proliferation	[183]	
	OSCC	IL-6	Increase CSC stemness	[103]	
	PC	IL-1b, IL-6, TNF-a	Increase CSC stemness	[102]	
	CRC	TNF-a	Increased CD4 + and CD8 + T cell infiltration	[171]	
M2-like macrophages	Melanoma	VEGF-A	Promote vascularization	[32]	
	Melanoma	PGE2, MMP-9	Promote vascularization, tumor growth and metastasis	[195]	
	LCA, PC	IL-6, TNF	Cauce inflammatory cell infiltration and carcinogenesis	[166, 167]	
	LCA	IL-6	Induce the EMT to enhance migration, invasion, and metastasis	[181]	
	LCA	ITG aVb3	Promote NSCLC metastasis	[184]	
	BC	IL-6	Support CSC survival and tumor progression	[168]	
	PC	IL-6	Promote cell proliferation, EMT and metastasis	[80, 196]	
	PDAC	HSP90a	Promote tumor growth	[187]	
	HCC	IL-6, TNF-a	Promote CSC stemness and EMT	[169]	
	HCC	MMP-9	Promote angiogenesis		
	HCC	miR-23a-3p	promote EMT, angiogenesis, and increase vascular permeability	[190]	
	CCA	TGF-b1	Promote the growth, EMT, and endoplasmic reticulum homeostasis	[185]	
	CRC	IL-6	Promote CRC cell proliferation and invasion	[188]	
	CRC	IL-10	Regulating M2 polarization; promoting tumor proliferation and migration	[76]	
	CRC	PDGF-BB	Promote CRC angiogenesis and its migration and invasion	[193]	
	CcRCC	CCL5	Promote tumor proliferation and migration	[186]	
	GBM	TGFBI	Promote tumor growth and GSC stemness	[182]	
	MM	IL-1, IL-18	Promote tumor growth	[189]	
	Ovarian cancer	IL-6	Increase CSC population	[191]	
	Ovarian cancer	CCL18	Promote EMT	[192]	

Abbreviations: HCC, hepatocellular carcinoma; OCSS, oral squamous cell carcinoma cell; LCA, lung cancer; PC, pancreatic cancer; BC, breast cancer; PDAC, pancreatic ductal adenocarcinoma; CCA, cholangiocarcinoma; CRC, colorectal cancer; CcRCC, clear cell renal cell carcinoma; GBM, glioblastoma; MM, multiple myeloma

In addition, TAMs are also known to produce an immunosuppressive TME by directly expressing cell surface proteins or secreting soluble factors, such as programmed death ligand 1 (PD-L1), sialic acid-binding Ig-like lectin 10 (siglec-10), arginase 1 (Arg1), indoleamine 2,3-dioxygenase (IDO), IL‑10, and TGF- [195–197]. These phenomena are closely associated with drug resistance observed in cancer immunotherapy. More detailed mechanisms of TAMs in the reshaping of TME will be discussed below.

**Fig. 2:**
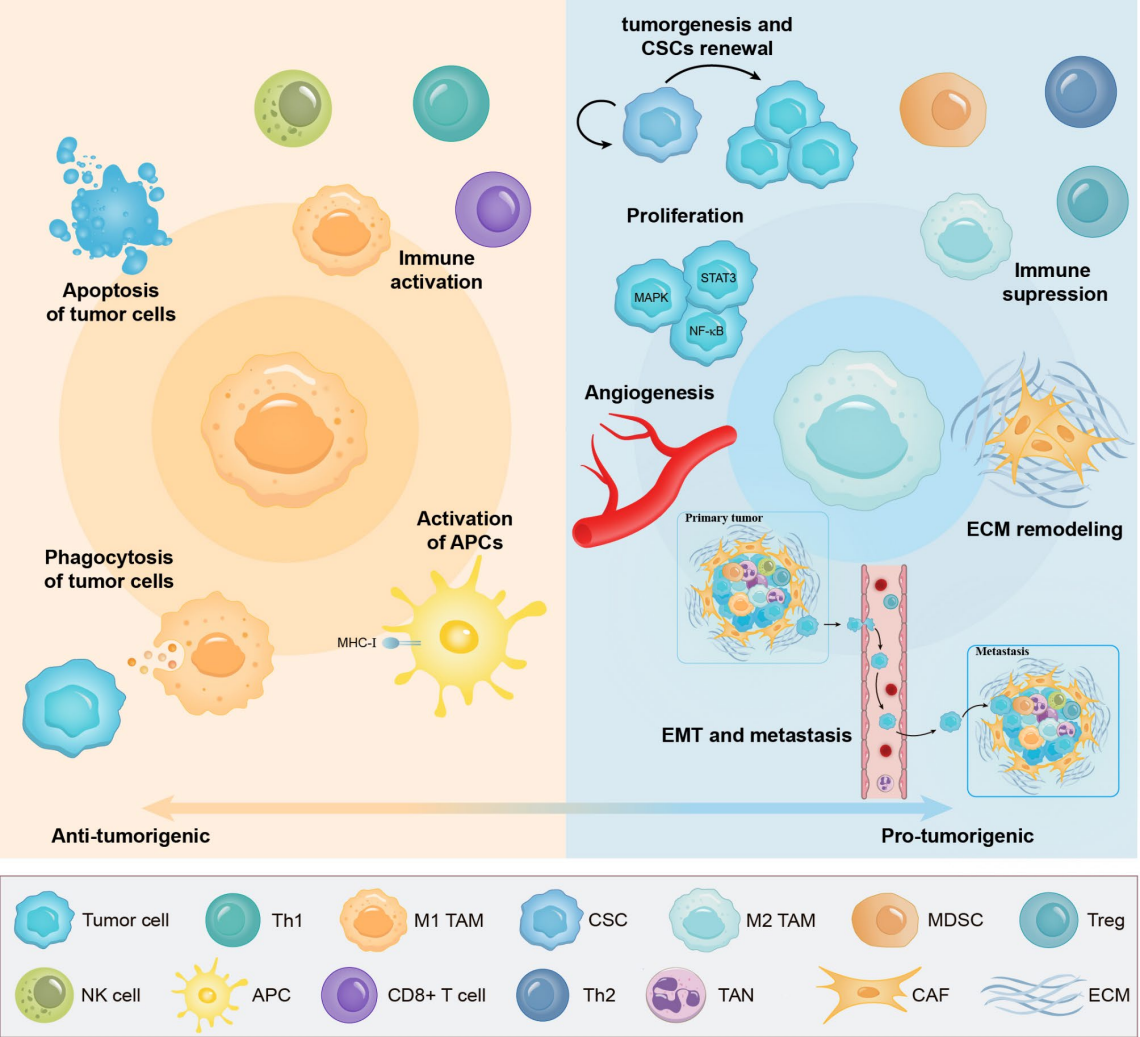
The phenotypes of TAMs and their dual roles in tumor progression. Macrophages have dual roles within the TME. Upon their recruitment to the TME, TAMs are believed to polarized into M1-type to restrict tumor progression (left-hand side), or differentiate towards M2-type TAMs to exert a pro-tumor role (right-hand side). M1-like macrophages are known to exert direct phagocytic and cytotoxic effects on tumor cells and induce their apoptosis. Furthermore, serving as the bridge between innate immunity and adaptive immunity, M1 macrophages stimulate the activation of antigen-presenting cells (APCs) and contribute to the immune activation by promoting the recruitment and functions of immunostimulant cells like CD8 + T cells, Th1 cells and NK cells. On the other hand, M2-like TAMs play key roles in cancer initiation and malignant progression by facilitating CSC renewal, stimulating proliferation, supporting tumor-associated angiogenesis, inducing epithelial-mesenchymal transition, favoring tumor cell distant metastasis, enhancing extracellular matrix (ECM) remodeling and suppressing the response to antitumor immunity

Collectively, TAMs are dynamic tumor promoters and immune suppressors throughout the various stages of tumor initiation and advancement via the expression of cell surface receptors and regulatory factors (Figure. 2).

### TAMs promote the formation of local immunosuppressive TME

**Fig. 3:**
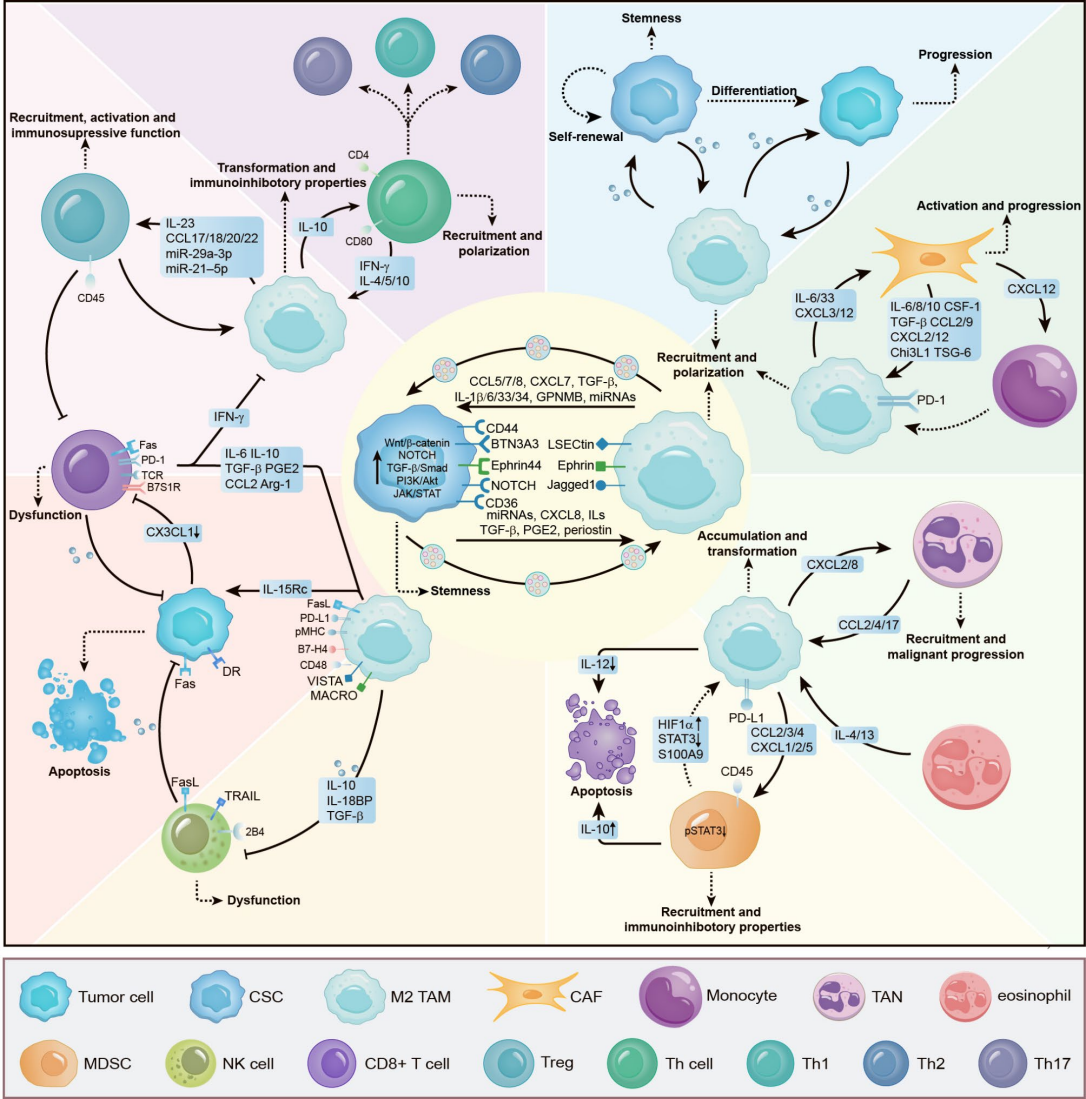
TAMs coordinate with other cellular components in the TME to mediate immunosuppression and therapeutic resistance. TAMs can maintain CSC stemness and endow tumor cells with therapeutic resistance through direct interplays, such as the secretion of MIF, GDF15, TGF-β, IL-1β, IL-6, IL-33, IL-34, CCL5, CCL7, CCL8, CXCL7, FN1, GPNMB, CHI3L1, miR-21, miR-223, miR-222-3p, miRNA-21-5p and the expression of cell surface protein like CD44, BTN3A3, Ephrin44, NOTCH and CD36. TAMs also interplay with other cells to orchestrate an immunosuppressive TME through the following pathways: (i) Promoting the trans-differentiation or polarization of other cells such as TAMs, TANs, CAFs, and T lymphocytes into certain pro-tumorigenic cell subsets; (ii) Recruiting and activating immunosuppressive functions including M2-type TAMs, TANs, Treg and MDSCs; (iii) Restraining the cytotoxic activity and cytokines production of effector immune cells including NK cells and CTLs. Collectively, these interactions reshape an immunosuppressive TME and are responsible for therapeutic tolerance

Clinically, a high TAM infiltration is significantly associated with unfavorable clinical outcomes for cancer patients with different tumor types. It is also responsible for the attenuated responses to standard-of-care therapeutics, including radiotherapy, chemotherapy, and immunotherapy [195, 198]. Numerous studies reported the significant role played by TAMs in shaping an inhibitory TME to mediate resistance to ICIs [17]. Recently, Wu et al. reported the upregulation of triggering receptor expressed on myeloid cell-1 (TREM-1) in TAMs by HIF-1α, which impaired the cytotoxic functions and induced apoptosis of CD8 + T cells in a PD-1/PD-L1-dependent manner. Besides, through the intricate CCL20/ERK/NF-κB signaling cascade, TREM-1 + TAMs also actively promoted the influx of CCR6 + Foxp3 + regulatory T cells (Tregs) into the TME to mediate immunosuppression [199]. To enhance the therapeutic efficacy of tumor treatment, it is important to understand possible mechanisms of intercellular communication involving TAMs within the TME (Figure. 3).

### Interaction between TAMs and tumor cells

The interaction between tumor cells and TAMs within the TME is closely associated with cancer drug resistance. Tumor cells are known to promote the infiltration and polarization of immunosuppressive M2 macrophages through various mechanisms mentioned above, which in turn endows the tumor cells with diminished sensitivity to anticancer drugs [200]. Cancer stem cells (CSCs) are a small subpopulation of malignant tumor cells capable of self-renewal and differentiation, which are associated with tumor initiation and progression [201]. The CSC and EMT phenotypes are significantly correlated with tumor invasion and metastasis [202]. More importantly, CSCs protect tumor cells from external assaults and orchestrate drug resistance through diverse mechanisms. Aldehyde dehydrogenase (ALDH) is a well-known CSC marker, which abrogates oxidative stress and imparts resistance to several chemotherapeutic drugs including platinum drugs [203]. On the other hand, enrichment of CSCs was found to be negatively correlated with tumor infiltration of effector T cells, which promoted immune evasion and dampened the efficacy of PD-L1 inhibitors [204]. Collectively, the stemness of CSC represents a major mechanism leading to drug resistance, tumor recurrence, and overall failure of tumor treatment strategies.

Recent research in CSC biology has revealed the significant role of TAMs in the interaction between CSCs and the TME. On the one hand, CSCs serve as a regulators for TAM infiltration and polarization. For instance, the activation of the Hippo pathway effector Yes-associated protein (YAP) is identified to determine the macrophages infiltration, which regulates oncogenic pathways including Kras, mTOR, β-catenin and promotes the recruitment immunosuppressive macrophages [205–207]. On the other hand, TAMs support CSC stemness and construct niches that are favorable for CSC survival to mediate drug tolerance. In PCA, CSCs have been shown to orchestrate the recruitment of macrophages into the TME and promote their differentiation to TAMs. Reciprocally, TAMs were reported to enhance the stem-like characteristics of CSCs and drug resistance via the IL-6/STAT3 signaling pathway [208]. Similar positive feedback loops are also indicated in IL1R2-overexpressing TNBC cells, highlighting the potential of IL1R2 blockade to reverse immunosuppression [209]. In CRC, ID1 expressing TAMs support cancer cell stemness and hinder CD8 + T cell infiltration. Therefore, the inhibition of TAM recruitment or immunosuppressive functions represent attractive strategies to attenuate CSC stemness and sensitize tumor cells to chemotherapy and immunotherapy [210, 211].

CSCs defend themselves against immune surveillance by secreting growth factors, metabolites, cytokines and other soluble substances (e.g., miRNAs, CXCL8, ILs, ECM, TGF-β, PGE2 and periostin) into the TME, which are positively correlated with macrophage infiltration and differentiation [212–215]. Chemokines like CCL5/8 and CXCL7 are shown to regulate the stem-like transition in PDAC and glioblastoma [216–218]. Interleukins secreted by TAMs also participate in the stemness of tumor cells. M2 TAMs could increase the number of CSCs in ovarian cancer through IL-6/ WNT5B axis [189]. Surprisingly, M1-like TAMs have also been shown to stimulate the JAK/STAT3 pathway by secreting IL-6, thereby increasing CSC stemness in oral squamous cell carcinoma cells, which indicates the protumor role of M1 and verifies the oversimplified binary classification again [103]. Recently, the IL-33-TGF-β niche signaling loop and IL-34-CD36 axis between CSCs and TAMs has been reported to promote immunosuppressive TAMs and therapeutic resistance in mouse models with squamous cell carcinoma or HCC with p53 inactivation [100, 219]. TAMs were also found to directly support tumor stemness by secreting other cytokines, such as the TGF-β1/smad2/3 pathway in pancreatic cancer [220] or the TGF-β1/integrin αvβ5/Src/STAT3 pathway and TGF-β2/smad2/3 in glioblastoma [180, 221], as well as MFG-E8/STAT3 and Hedgehog signals in lung cancer [222]. GPNMB is a pro-inflammatory glycoprotein highly expressed in macrophages and microglia, which could be cleaved by proteases. In mouse tumor models, M2 TAMs have been shown to preferentially express soluble GPNMB which combines with the CD44 receptor on tumor cells and promotes CSC proliferation via PI3K/AKT/mTOR or β-catenin/MAPKs/AMPK/Src signaling [223]. Besides, TAM-derived extracellular vesicles were also shown to mediate tumor invasion. For instance, M2 macrophage-derived exosomal microRNA-21-5p was reported to induce differentiation and activity of pancreatic CSC by targeting the transcriptional repressor Kruppel-like factor 3 (KLF3) [224]. Contact-dependent interactions based on the expression of cell surface protein on TAMs which drive cancer stemness include the binding of Jagged1 to NOTCH on liver CSCs, the binding of LSECtin to BTN3A3 as well as the binding of Ephrin to Ephrin type A on mammary stem cells [225–227]. Taken together, these results indicate that TAMs play a crucial role in the maintenance of CSC stemness mainly through the secretion of effector molecules like chemokines, cytokines, proteins and miRNAs and the contact-dependent interplays.

### Interaction between TAMs and CAFs

CAFs are pivotal components of the TME and they play critical roles in promoting cancer angiogenesis and metastasis, reshaping the extracellular matrix, promoting an immunosuppressive TME, and inducing drug resistance [228]. CAFs generally originate from tissue-resident fibroblasts and other normal cells through various pathways. It is noteworthy that TAMs are usually abundantly found around the CAF settlement area, suggesting tight interactions between the two cell types [229]. CAFs were shown to interact with TAMs to maintain an inhibitory TME and constrain immunotherapy efficacy, thereby causing dismal prognosis in cancer patients [230, 231].

Cumulative evidence showed that CAFs promote the recruitment of TAMs to the TME and macrophage polarization towards the pro-tumorigenic phenotype (i.e., M2-type TAMs) via various regulatory molecules, including IL-6/8/10/34, CSF-1, TGF-β, CCL2/9, CXCL2/12, chitinase 3-like 1 (Chi3L1), TNF-α, TNF-Stimulated Factor 6 (TSG-6) and expression of endosialin [99, 232–237]. Collectively, CAFs impair antitumor response from effector T cells and elicit immune suppression in the TME [238]. This is exemplified by the involvement of CAFs in the formation of a pro-metastatic niche in hepatocytes, TAM accumulation and liver metastasis via an IL6/STAT3/SAA pathway [239].

More importantly, CAFs are capable of inducing the immunoinhibitory properties of TAMs. After migrating to the tumor site via the CAF-driven CXCL12-CXCR4 axis, monocytes/macrophages may transform into M2-type TAMs in a HIF2-dependent manner to induce an immunosuppressive microenvironment and mediate resistance to ICIs [45, 240]. Consistently, combined MEKi + STAT3i that endows CAFs with mesenchymal stem cell (MSC)-like properties can reprogram M2-like macrophages towards M1-type, verifying the partial contribution of CAFs in TAM polarization [241]. Recently, Gordon et al. reported the CAF-induced upregulation of programmed cell death protein 1 (PD-1) on M2-type TAMs, which was linked to the suppression of both innate and adaptive antitumor immune response [242, 243]. On the other hand, M2-type macrophages can regulate CAF activation and progression. Comito et al. [244] reported that M2-type macrophages could stimulate CAF activation by secreting soluble factors, including IL-6 and SDF-1. Subsequently, activated CAFs can further enhance TAM activity. In PDAC mouse models, a CAF-to-myoCAF could be mediated by IL-33-ST2-MYC-CXCL3-CXCR2 axis or JAK/STAT signaling activated by macrophage-derived progranulin and cancer cell-secreted leukemia inhibitory factor (LIF). Reciprocally, myoCAFs promoted tumor metastasis and released osteopontin to improve an immunosuppressive macrophage phenotype and T cell dysfunction [245, 246]. Utilizing scRNA-seq and spatial analysis, similar spatial networking around SPP1 + macrophages and CAFs, including FAP + CAFs, was also indicated in CRC, metastatic colorectal cancer (mCRC) liver tumor and other solid tumors [231, 247, 248]. It seems that disrupting SPP1 + macrophages and fibroblasts communication represents a promising strategy to boost immunotherapy.

### Interaction between TAMs and immune cells

#### Interaction between TAMs and MDSCs

Myeloid‑derived suppressor cells (MDSCs) are immature myeloid cells present in circulation and TME. Unlike the orderly maturation process under physiological conditions, immature myeloid cells deviate from the normal differentiation trajectory when exposed to continuous inflammatory signals in pathological conditions such as chronic inflammation and cancer [249]. These cells lack unique and identifiable surface markers and are characterized by an immature phenotype, morphology, relatively weak phagocytic activity, and immunosuppressive function [250]. MDSCs are divided into two subtypes: monocytic MDSCs (M-MDSCs), which resemble monocytes, and granulocytic or polymorphonuclear MDSCs (G-MDSCs/PMN-MDSCs), which are similar to neutrophils [251]. The former is characterized by CD11b + Ly6G − Ly6C^hi^ in mice and CD11b + CD14 + HLA-DR−/loCD15 − in humans, while the latter exhibits CD11b + Ly6G + Ly6C^lo^ in mice and either CD11b + CD14 − CD15 + or CD11b + CD14 − CD66b + in humans [252]. Currently, MDSCs and TAMs are primarily distinguished by their functions and surface markers. Several novel surface markers have been proposed to differentiate M-MDSCs from TAMs, such as CD11b + Ly6G − Ly6C^hi^CD84 + in mice and CD14+/CD66b − CXCR1 + or CD14+/CD66b − CD84 + in humans [253]. However, both MDSCs and TAMs exhibit significant heterogeneity and plasticity, underscoring the need for more detailed and comprehensive classification methods.

MDSCs could be differentiated into TAMs. The differentiation of TAMs in tumor site was controlled by the downregulation of STAT3 transcription activity. Under hypoxic condition in tumor sites, the upregulation of CD45 tyrosine phosphatase activity in MDSCs is needed to mediate the downregulation of STAT3 in a HIF-1α-independent manner [254]. Likewise, transcription factor C/EBPβ is activated by S100A9 to trigger M2 differentiation, which may augment the expression of PD-L1 on TAMs and induce immunosuppression [225, 255]. This seems to provide a chance of the administration of TAM-targeted therapy. However, in a murine model of CCA, the blockade of TAMs with anti-CSFR leads to a compensatory accumulation of ApoE G-MDSCs with immunosuppressive signatures. In this context, dual inhibition of G-MDSCs and TAMs potentiates anti-PD-1herapy, indicating the importance to unveil the interactions in TME and design combination therapy with caution [256]. Meanwhile, MDSCs also facilitate immunosuppression through a direct interplay with other immune inhibitory cells [257]. It has been reported that MDSCs tend to differentiate into M2-type TAMs and elicit a type 2 tumor-promoting immune response [257]. The increased secretion of IL-10 from MDSCs but reduced production of IL-12 from macrophages were shown to promote T-cell apoptosis, thereby restricting the immune response [258]. However, the relationship between TAMs and MDSCs has not been fully elucidated. Recent researches have proved that TAM-derived chemokines like CCL2/3/4, CXCL1/2/5 modulates the proliferation, recruitment, and immunosuppressive functions of MDSCs [259, 260]. On the contrary, TAMs may also suppress the recruitment of MDSCs to the tumor or inhibit the T cell immune response directly by elevating expression of the immunosuppressive ligand PD-L1, thus providing a novel target for cancer immunotherapy [261].

#### Interaction between TAMs and TANs

Tumor-associated neutrophils (TANs) were shown to regulate tumor cell adhesion to endothelial cells and the subsequent migration to metastatic sites [26]. The crosstalk between TANs and TAMs, via chemokines and cytokines, play a critical role in inducing inhibitory TME, hampering antitumor immunity and supporting tumor progression [44]. CCL2 and CCL17 secreted by TANs and peripheral blood neutrophils were shown to increase the number of macrophages recruited to the TME, which is associated with the progression of HCC and resistance to sorafenib [262]. Another recent study also shown that CCL4 + TANs could recruit macrophages with unknown phenotypic characteristics [263]. TAMs are believed to suppress the antitumor immunity and promote tumor development by similar machineries. It has also been reported that neutrophil accumulation in the TME was mediated by an increased CXCL2 and CXCL8 via the PFKFB3-NF-κB pathway [264]. As there are only limited reports about the interaction of TAMs and TANs, other specific mechanisms underlying the mutual effects of TAMs and TANs on each other remain to be explored.

#### Interaction between TAMs and eosinophils

As integral components of innate immunity, eosinophils are shown to infiltrate various tumors and exhibit dual roles in tumor progression [265]. Intriguingly, these cells can exert anti-tumor effects by releasing mediators such as CXCL9, CXCL10, TNF-α, granzyme, cationic proteins, in melanoma, CRC, and HCC [265]. Conversely, they can also adopt pro-tumor functions through the secretion of pro-angiogenic factors and growth factors [266]. Similarly, their interactions with tumor-associated macrophages (TAMs) display contrasting outcomes. On one hand, eosinophils directly attack tumor cells via eosinophil peroxidase (EPX) or eosinophil-derived neurotoxin (EDN), or indirectly stimulate macrophages to release TNF-α and H_2_O_2_. Furthermore, eosinophils promote TAM polarization toward anti-tumor M1-like phenotypes via CXCL9 secretion, which recruits CD8 + T cells to bolster immune checkpoint inhibitor (ICI) efficacy. This process also enhances vascular normalization and reinforces M1 polarization [267, 268]. On the other hand, eosinophils paradoxically drive immunosuppression by facilitating M2-like TAM polarization. Specifically, IL-4 and IL-13 secreted by eosinophils regulate M2 polarization, a process counteracted by TNF receptor signaling pathways [269]. Given these dual roles, the impact of eosinophils on ICI therapeutic outcomes warrants further investigation.

#### Interaction between TAMs and NK cells

Natural killer (NK) cells are lymphocytes generated from the hematopoietic stem cells in the bone marrow. They can directly kill tumor cells without the need to recognize a tumor-specific surface antigen [270], thus making NK cells an attractive effector for cancer immunotherapy. Activation of NK cells is tightly regulated by the balance of activating and inhibitory signals. When the activating ligands are upregulated (i.e., induced-self) or inhibitory ligands are downregulated (i.e., missing-self) in the target cells, NK cells will be engaged to kill the targets via secretion of perforin and granzymes, the production of interferon-γ (IFNγ), or expression of the death ligands such as FASL and TRAIL. While the knowledge about interactions between NK cells and TAMs is limited, numerous studies demonstrated that TAMs regulated the antitumor cytotoxicity of NK cells. First, TAMs are known to indirectly inhibit NK cells via secretion of various cytokines. Both Upregulated IL-10 and downregulated IL-15 and CXCL10 are known to suppress NK cell activities [271–274]. Moreover, TAMs isolated from the metastatic lung were shown to suppress NK cell-induced tumor cell apoptosis in vitro in a TGF-β-dependent manner [275]. Contact-dependent interplays also exit between NK cells and TAMs. The expression of VISTA and MACRO on TAMs is negatively correlated with NK cell infiltration. The inhibition of VISTA and MACRO is shown to contribute to more NK cell infiltration and the activation of NK cell killing in a TRAIL-dependent manner, which can synergize with ICI-based therapies against tumors [272, 276]. In HCC, CD48-expressing TAMs were shown to interact directly with the CD48 receptor 2B4 on NK cells and lead to NK cell dysfunction [277]. On the other hand, TAMs may also improve their functions. It has been proven that the stimulation of STING-type I IFN signaling promotes the activation of NK cells and improves the efficacy of ICIs [278, 279]. At the same time, IFN-γ secreted by activated NK cells is essential for TAM accumulation [280]. In addition, STING activation can also enhance NLRP3-mediated IL-18 and IL-1β secretion from TAMs to promote 4-1BBL/4-1BB-dependent NK cell antitumor cytotoxicity [281]. A recent study indicated that TREM2 macrophages not only secreted IL-18 BP to inhibit the impact of IL-18 on NK cells but also alleviated the DC-derived IL-15, which drives NK cell dysfunction in lung cancer and provides a deeper insight into dual targeting of NK cells and TAMs [107]. To date, the interactions between TAM and NK cells have been scarcely investigated. More studies are warranted to understand whether they are involved in the dysfunction of NK cells during tumor immune escape.

#### Interaction between TAMs and CD4 + T cells

T lymphocytes play a critical role in adaptive immune response. There are different subtypes, including Treg cells, helper T (Th) cells, and cytotoxic T lymphocytes (CTLs) [282]. By conducting scRNA-seq analysis on metastatic lung cancer biopsies from cancer patients before and during targeted therapy, Maynard et al. reported over 20,000 cancer and TME single-cell profiles illustrating rich and dynamic tumor ecosystem [283]. Interestingly, active T-lymphocytes but decreased macrophages were present at residual disease whereas immunosuppressive cell states were present at progressive disease [283], suggesting a link between TAMs and T cells.

T helper (Th) cells are known to affect the polarization and function of macrophages. IFN-γ released by Th1 cells generates M1 macrophages whereas IL-4, IL-5, and IL-10 secreted by Th2 cells induce M2 polarization [284]. PD-L1 and PD-L2 are ligands for PD-1, which plays an inhibitory role in regulating T-cell activation. Loke et al. reported that PD-L1 is highly inducible on various antigen-presenting cells as well as resident macrophages but PD-L2 is inducible only on inflammatory macrophages. Consistently, Th1 cells and microbial products can upregulate PD-L1 expression on various macrophage populations whereas Th2 cells could only instruct inflammatory macrophages to upregulate PD-L2 [285]. Therefore, PD-L1 and PD-L2 exhibit different functions in regulating type 1 and type 2 immune responses. To this end, the co-culture of naïve CD4 + T cells with PD-L1-positive TAMs was found to induce the expression of CD80 on CD4 + T cells, which mediated the resistance to anti-PD-1 and anti-CTLA-4 therapies in glioma cells [286]. On the other hand, macrophages also determine the fate of Th cells infiltration, differentiation and function. In glioblastoma, TAMs were demonstrated to restrain T cell infiltration and activation in a Siglec-9-dependent manner [287]. In pancreatic ductal adenocarcinoma, TAMs were shown to induce Th2 cells, Th17 cells, and Tregs polarization via a IL-10/NOD-like receptor family pyrin domain–containing 3 (NLRP3) pathway, whereas they retarded Th1 polarization and cytotoxic T cells activation [288]. A recent study pointed out that T cell-derived IFN-γ induced TAM differentiation towards M1-like phenotype, which would reciprocally remodel TME to favor T cell infiltration and immune function [289].

CD4 + CD25 + Foxp3 + regulatory T (Treg) cells represent the major immunosuppressive cell population in the TME to preserve immune homeostasis [108]. In general, TAMs and Tregs work synergistically to enhance immunosuppression and mediate immune evasion and ICI resistance [290]. On the one hand, M2-type TAMs are known to secrete various chemokines, including CCL17, CCL18, and CCL22, to promote the recruitment of Treg cells and restrain the activation of cytotoxic T cell [291, 292]. Specifically, the infiltration of TREM-1 + TAMs in HCC were shown to mediate hypoxia-induced tumor immunosuppression and resistance to anti-PD-L1 therapy, by recruiting CCR6 + Foxp3 + Treg cells via the CCL20/ERK/NF-κB pathway [199]. Recently, Zhou et al. reported that TAM-derived exosomes enriched in miR-29a-3p and miR-21–5p could directly suppress T cell-intrinsic STAT3 and regulate Treg/Th17 in ovarian cancer [293]. Unexpectedly, IL-23 from TAMs stabilized Tregs and enhanced their immunosuppressive impact on cytotoxic T cells to mediate immune evasion in preclinical models, making IL-23/IL-23R axis a new means of eliciting antitumor responses [294, 295]. On the other hand, the recruited Treg cells can further modulate the extent of immunosuppression of TAMs. Tregs are known to transform monocytes toward M2 TAMs and limit CD8 + T cells-derived IFN-γ to regulate metabolic processes and sustain their survival [291, 296]. Collectively, there is a positive feedback loop between TAMs and Tregs that reinforces their immunosuppressive effects within the TME.

#### Interaction between TAMs and CD8 + T cells

CD8 + cytotoxic T cells are considered the most critical effectors of antitumor immunity, which are able to induce apoptosis of tumor cells [229]. Therefore, the inactivation of CD8 + T cells can lead to immunotherapy resistance.

Kuppfer cells (KCs) are resident macrophages of the liver and they play key roles in liver immunity. KCs are known to protect against HCC by communicating with other immune cells. M2 polarization of KCs has been shown to induce HCC development in mouse model. Recently, Liu et al. reported that microRNA-206 could promote the recruitment of CD8 + T cells by driving M1 polarization of KCs via a KLF4-CCL2 pathway, suggesting its potential use as an immunotherapeutic approach [73]. Importantly, TAMs can regulate the tumor cell-killing ability of cytotoxic T cells via direct contact or secretion of soluble factors. High expression of co-inhibitory molecules expressed on TAMs, such as PD-L1 and B7-H4, was shown to suppress CD8 + T cells checkpoint inhibitor (ICI) therapy in various tumors [48, 297, 298]. In HCC, TAMs can also eliminate antitumor CD8 + cytotoxic T cells via Fas-dependent apoptosis [299]. On the other hand, several cytokines and metabolites released by TAMs, including IL-6, IL-10, TGF-β, PGE2 and Arg1, are known to cause cytotoxic T-cell dysfunction [48, 300]. Moreover, TAMs also regulate T-cell activity indirectly. For instance, IL-15Rc (i.e., IL-15/IL-15Rα complex) has been shown to impede chemokine CX3C chemokine ligand 1 (CX3CL1) secretion by breast cancer cells, subsequently restricting the infiltration and activation of T cells [301].

### Strategies to target TAMs

Through regulating the functions of tumor cells and other cells in TME, the complex role of TAMs is tightly linked to tumor progression and antitumor immune responses. Interestingly, the dual functions and remarkable plasticity of TAMs have provided ample motivation and opportunities for TAM-targeted strategies to fight cancer [302]. Such strategies have been proved to produce synergized antitumor effects with existing agents and have achieved impressive treatment efficacy in animal models [303, 304]. Several drugs targeting TAMs in combination with ICIs are also being tested in clinical trials, which is outlined in Table 4.

**Table 4:** Ongoing clinical trials evaluating the combination of TAM-targeted therapy and ICIs

Strategies	Agent	Cancer type	Combination partners	Clinical trials registry	Clinical phase
Macrophages recruitment inhibition	CSF1/CSF1R inhibitor				
	Vimseltinib (DCC-3014)	Sarcoma	Avelumab	NCT04242238	I (Active)
	Axatilimab (SNDX6352)	Solid tumor	Retifanlimab + chemotherapy	NCT06320405	I/II (Recruiting)
		HL	Nivolumab	NCT05723055	II (Recruiting)
		TNBC	Pembrolizumab	NCT05491226	II (Recruiting)
	Cabiralizumab	TNBC	Nivolumab + chemotherapy	NCT04331067	I/II (Active)
		HCC	Nivolumab	NCT04050462	II (Active)
Macrophages elimination	Trabectedin	Sarcoma	Nivolumab	NCT03886311	II (Recruiting)
Macrophages reprogram	PI3Kg inhibitor				
	IPI-549	TNBC, RCC	Atezolizumab + bevacizumab /nab-paclitaxel	NCT03961698	II (Active)
	BYL719	Solid tumor	Atezolizumab/ ipilimumab/ nivolumab	NCT04591431	II (Active)
	Duvelisib (IPI-145)	Melanoma	Nivolumab	NCT04688658	I/II (Active)
	STAT3 inhibitor				
	AZD9150	NSCLC	Durvalumab + chemotherapy	NCT03421353	I (Active)
		NSCLC	Durvalumab + danvatirsen	NCT03819465	I (Active)
		Bladder cancer	Durvalumab	NCT02546661	I (Active)
		Solid tumors, HNSC	Durvalumab + tremelimumab	NCT02499328	I/II (Active)
		NSCLC	Durvalumab	NCT03334617	II (Active)
		PCA, NSCLC, CRC	Durvalumab + danvatirsen	NCT02983578	II (Active)
	TLR agonist				
	MGN1703	Solid tumor	Ipilimumab	NCT02668770	I (Active)
	CMP-001	Lymphoma	Pembrolizumab	NCT03983668	I/II (Active)
	TransCon TLR7/8 Agonist	Solid tumor	Pembrolizumab	NCT04799054	I/II (Active)
	TransCon TLR7/8 Agonist	HNSC	Pembrolizumab	NCT05980598	II (Active)
	BDC-1001	HER2 + Solid tumor	Nivolumab	NCT04278144	I/II (Active)
	CD40 agonist antibody				
	Selicrelumab (RO7009789)	PDAC	Atezolizumab + chemotherapy	NCT03193190	I/II (Active)
		BC	Atezolizumab + bevacizumab	NCT03424005	I/II (Recruiting)
	CDX-1140	Solid tumor	TCR-T + pembrolizumab	NCT04520711	I (Active)
	APX005M	Melanoma	Pembrolizumab	NCT02706353	I/II (Active)
		Melanoma, RCC	Nivolumab + Ipilimumab	NCT04495257	I (Active)
		PC	Zimberelimab + domvanalimab	NCT05419479	I/II (Recruiting)
		PC, CRC	Pembrolizumab	NCT02600949	I (Recruiting)
		Ovarian cancer	Pembrolizumab + bevacizumab	NCT05231122	I/II (Recruiting)
	IL-4R inhibitor				
	Dupilumab	NSCLC	Cemiplimab	NCT06088771	I/II (Recruiting)
	IL-6/IL-6R inhibitor				
	Tocilizumab	Melanoma	Ipilimumab + Nivolumab	NCT03999749	II (Active)
		Melanoma, Urothelial carcinoma, NSCLC	Ipilimumab + Nivolumab	NCT04940299	II (Active)
		PCA	Atezolizumab	NCT03821246	II (Active)
		GBM	Atezolizumab	NCT04729959	II (Active)
		PDAC	Atezolizumab + Gemcitabine + Nab-Paclitaxel	NCT03193190	I/II (Active)
		NSCLC	Atezolizumab	NCT04691817	I/II (Recruiting)
		Liver cancer	Atezolizumab + Bevacizumab	NCT04524871	I/II (Recruiting)
		HNSCC	Atezolizumab	NCT03708224	II (Recruiting)
	Siltuximab	Solid tumor	Anti-PD-L1	NCT06470971	II (Recruiting)
	Sarilumab	Melanoma	Ipilimumab + Nivolumab/Relatlimab	NCT05428007	I/II (Recruiting)
		NSCLC	Cemiplimab	NCT05704634	I (Recruiting)
		Ovarian cancer	REGN4018 + cemiplimab	NCT03564340	I/II (Recruiting)
	IL-8/IL-8R inhibitor				
	BMS-986,253	HCC	Nivolumab	NCT04050462	II (Active)
		NSCLC, HCC	Nivolumab	NCT04123379	II (Active)
		Solid tumor	Ipilimumab + Nivolumab	NCT03400332	I/II (Active)
		Solid tumor Melanoma	Nivolumab	NCT04572451	I (Recruiting)
		HNSCC	Nivolumab	NCT04848116	II (Recruiting)
		PDAC	Nivolumab	NCT02451982	II (Recruiting)
		CRC	Nivolumab	NCT03026140	II (Recruiting)
	AZD5069	Solid tumor HNSCC	MEDI4736	NCT02499328	I/II (Active)
Macrophages phagocytosis	AntiCD47 antibody				
	Magrolimab (Hu5F9-G4)	HL	Pembrolizumab	NCT04788043	II (Active)
		Urothelial carcinoma	Atezolizumab	NCT03869190	I/II (Recruiting)
	Golcadomide (CC-9002)	Follicular lymphoma	Nivolumab + rituximab	NCT05788081	II (Recruiting)
	Evorpacept (ALX148)	Solid tumors, lymphoma	Pembrolizumab	NCT03013218	I (Active)
		CRC	Cetuximab + pembrolizumab	NCT05167409	II (Active)
		HNSC	Pembrolizumab	NCT04675294	II (Active)
		HNSC	Pembrolizumab	NCT04675333	II (Active)
		Ovarian cancer	Pembrolizumab + doxorubicin	NCT05467670	II (Recruiting)
	SIRP- Fc mAb				
	TTI-621	DLBCL	Pembrolizumab	NCT05507541	II (Recruiting)
	AntiCD47/PD-1 antibody				
	HX009	Solid tumors	None	NCT05731752	I (Active)
		Lymphoma	None	NCT05189093	I/II (Recruiting)

Abbreviations: PDAC, pancreatic ductal adenocarcinoma; HL, hodgkin lymphoma; TNBC, triple negative breast cancer; HCC, hepatocellular carcinoma; RCC, renal cell carcinoma; NSCLC, non-small cell lung cancer; HNSC, head and neck squamous cancer; CRC, colorectal cancer; PCA, prostate carcinoma; BC, breast cancer; PC, pancreatic cancer; DLBCL, diffuse large B-cell lymphoma

#### Inhibition of macrophage recruitment to the TME

As discussed above, the recruitment of TAMs relies heavily on several chemokine signals. Therefore, the relevant effector molecules of these chemokine signaling pathways are promising therapeutic targets. CCL2/CCR2 signaling exerts a central effect on the recruitment of TAMs to the TME, making it feasible to explore relevant treatment strategies [305]. To this end, CCL2-neutralizing antibody or CCR2 antagonists (PF-04136309, RS504393, CCX872) have been used to interrupt CCL2/ CCR2 signaling, reduce the number of TAMs recruited to TME and potentiate the antitumor efficacy of ICIs in experimental animal models [306–309]. However, these anti-CCR2 antibodies and small-molecule inhibitors only yielded marginal therapeutic efficacy when used alone or in conjunction with chemotherapy or immunotherapy in clinical trials probably because of the massive redundancy of the chemokine system [310, 311]. In mouse models of breast cancer, interruption of CCL2/CCR2 signaling leads to acceleration of tumor metastasis as a result of massive release of monocytes [312], indicating that more potential compensatory factors should be considered to achieve optimal and durable effects when exploring TAM-targeted strategies.

CSF1/CSF1R targeted therapy is another promising strategy to inhibit macrophage recruitment to the TME. PLX3397 (also called pexidartinib) is an oral and potent multi-targeted receptor tyrosine kinase inhibitor of CSF-1R, c-Kit and FLT3. It has been shown to remarkably reduce the viability of M2 macrophages but it has no impact on M1 macrophages. When used in combination with rituximab (monoclonal anti-CD20 antibody) or DC vaccination, PLX3397 synergistically enhanced the overall patient survival with decreased TAMs and increased immune responses to immunotherapy [313, 314]. It is noteworthy that inhibition of CSF-1R also promotes the reprogramming of TAMs towards the M1 phenotype [314]. Since CSF-1/CSF1R blockade may interfere with Tregs and DCs in the TME, the use of CSF1R inhibitors may also trigger compensatory effects, induce other pro-survival pathways and create limited outcomes [16, 315]. In cancer patients, CSF1-R inhibitors, including small-molecule agents (vimseltinib) and monoclonal antibodies (axatilimab, emactuzumab, cabiralizumab) are now being tested in combination with ICIs.

#### Elimination of macrophages

The clearance of TAMs from the TME is obviously a direct method to overcome immunosuppression. Zoledronic acid (ZA) was shown to be actively taken up by macrophages and caused significant depletion of TAMs. ZA plus thymosin α1 or anti-PD-L1 therapy was found to significantly relieve immunosuppression in PCA cells or HCC, stimulate pro-inflammatory macrophages, and activate cytotoxic T cells [316–318]. Trabectedin is an antitumor chemotherapeutic drug that was also shown to selectively kill monocytes in the circulation and TAMs in tumors via a TRAIL-dependent mechanism [319]. Recently, the TAMs-mediated antitumor efficacy of trabectedin has been reported in preclinical models of PCA, and ovarian cancer [320, 321].

#### Macrophage reprogramming

While the vast majority of TAMs exhibit pro-tumor effects, TAMs display a high degree of plasticity within the TME and they are also capable of antigen presentation, phagocytosis, and triggering cytotoxic T cell responses following relevant stimulation [22]. Therefore, compared with inhibition of macrophage recruitment or depletion, reprogramming of macrophages to adopt an “immune-supportive” phenotype has emerged as an alternative strategy for antitumor therapy.

##### PI3K signaling pathway

The immunosuppressive properties of TAMs are generally believed to be dependent on the phosphatidylinositol 3-kinase γ (PI3Kγ) signaling. Consistently, the combination of PI3K signaling-targeted therapy with vasculature disrupting agents, other kinase inhibitors, or checkpoint inhibitors have produced different extent of enhanced antitumor effects [322–324]. Alpelisib (BYL719) is a potent and selective PI3Kα inhibitor clinically approved for the treatment of HR+/HER2-, PIK3CA-mutated, and advanced breast cancer. PIK3CA mutation is a key predictor of the response to PI3K inhibitors [325]. Duvelisib (IPI-145), a dual PI3Kδ/γ inhibitor, exhibits profound antitumor efficacy for the treatment of chronic lymphocytic leukemia (CLL) or small lymphocytic lymphoma (SLL) [326]. It also produces a synergistic antitumor effect with venetoclax for the treatment of Richter Syndrome, CLL, or SLL [327].

Recently, KTC1101, a novel pan-PI3K inhibitor has been shown to cooperate with anti-PD-1 therapy by restricting tumor proliferation and improving antitumor immune responses [328]. Clinical trials are now in progress to explore more favorable treatment combinations.

##### STAT3 signaling

In immune cells, signal transducer and activator of transcription 3 (STAT3) signaling is usually associated with tolerogenic immune response. Therefore, STAT3 is considered an attractive target for cancer therapy [329]. To this end, the STAT3-specific antisense oligonucleotide AZD9150 (also known as danvatirsen) was shown to produce a synergistic antitumor effect with immune checkpoint inhibitors (ICIs) in several tumor models [330], including the ICI-resistant tumor models caused by deletion of the tumor suppressor STK11 [331]. Of note, STK11 deletion is known to significantly reduce the infiltration of NK cells and inhibit the viability and chemotactic ability of NK cells. To date, several selective STAT3 inhibitors have been developed including small-molecule inhibitors (napabucasin, TTI-101, OPB-51602, OPB31121, OPB-111077, BP-1-102, and S3I-201) and antisense oligonucleotides (danvatirsen/AZD9150 and STAT3 DECOY) [329, 332, 333].

##### TLRs signaling

As mentioned above, the activation of Toll-like receptors (TLRs) by pathogen-associated molecular patterns (PAMPs) and danger-associated molecular patterns (DAMPs) has been proven to convert M2-like TAMs into M1-type and restrict immunosuppression, such as TLR3, TLR4, TLR7/8, and TLR9 [334–336]. Surprisingly, TLR stimulation in TAMs coexists with the increased expression of PD-L1, which attenuates the cytotoxic effects of CD8 + T cells. This adverse effect seems to provide feasibility for the combined application of TLR agonist and PD1 inhibitor, such as TLR7 agonist 1V270 in head and neck squamous cell carcinoma [337], TLR5 agonist KMRC011 in CRC [112]. A completed Phase I study (*NCT03301896*) has also explored the feasibility and safety of LHC165, a TLR7 agonis, both alone (*n* = 20) and in combination with PD-1 blockade spartalizumab (*n* = 19) in patients with advanced solid tumors [338].

##### CD40 and its ligands

CD40 is a transmembrane protein present on macrophages, B cells, and follicular dendritic cells. CD40-CD40L interactions stimulate antigen-presenting cells-derived IL-12, subsequently promoting antitumor T cell activation, B lymphocyte differentiation, and antibody secretion. The combination of CD40 agonist antibody and anti-CSF-1R therapy was shown to prime the macrophages to polarize towards the proinflammatory phenotype, thereby triggering potent T cell responses even in tumors nonresponsive to ICIs [339–341]. Following the combination of CD40 agonist monoclonal antibody and ICIs, significant tumor regression was observed in animal models with various tumor types like PDAC, bladder cancer and cholangiocarcinoma [342–344]. Currently, CD40 agonist mAbs are under clinical investigation in combination with ICIs or chemotherapy in patients with advanced solid tumors [345].

##### ILs and their receptors

Given the dynamic role of interleukins in macrophage polarization and TME restoration, modulating their activity—either by enhancing pro-inflammatory signals or suppressing immunosuppressive pathways—represents a promising strategy in immunotherapy. Preclinical studies have validated the synergy between M1-polarizing interleukins and ICIs. For example, Dipongkor Saha et al. reported that combining anti-CTLA-4, anti-PD-1, and G47Δ-mIL12 in glioma models significantly enhanced M1-like polarization and elevated the ratio of effector T cells to regulatory T cells [346]. Similar effects have also been observed in other M1-polarity interleukins like IL-2 [347].

Indeed, the blockade of M2-polarity interleukins has gained more attention due to their immunosuppressive functions. IL-6, for instance, is known to drive TAM polarization toward M2-like phenotypes via JAK/STAT signaling. Blocking IL-6 not only induces immunogenic cell death (ICD) in tumor cells but also reprograms TAMs toward the M1 phenotype, effectively overcoming ICI resistance while minimizing immune-related adverse events [348–350]. IL-6/IL-6R antagonists (e.g., tocilizumab, siltuximab) are currently being evaluated in clinical trials in combination with ICIs. Similarly, as a prognostic biomarker in ICI-treated cancer patients, IL-8 has emerged as another promising target for TAM repolarization and ICI efficacy enhancement [351, 352]. Clinical trials assessing the efficacy and safety of IL-8 inhibitors (e.g., BMS-986253 and AZD5069) combined with Nivolumab are underway across multiple cancer types. Recently, the immunosuppressive effect of IL-33 has attracted much attention. Targeting IL-33/ST2 signaling reprograms TME and potentiates anti-PD-L1 immunotherapy responses in lung cancer, melanoma and CRC [98, 353, 354]. While these findings underscore therapeutic potential, further clinical trials are needed to assess the safety and applicability of IL-33/ST2-targeted strategies.

#### Macrophage phagocytosis

Macrophages play critical roles in antibacterial and anti-tumoral responses by engulfing foreign xenobiotics, waste products, aging cells and tumor cells. In physiological conditions, normal cells evade macrophage-mediated clearance through the expression of anti-phagocytic molecules, termed phagocytosis checkpoints [355]. Tumor cells exploit similar evasion strategies by overexpressing these inhibitory signals (e.g., CD47, CD24) or reprogramming macrophages toward pro-tumoral phenotypes via tumor microenvironment (TME) remodeling [355–357]. Therefore, therapeutic strategies targeting phagocytic checkpoints, antibody-dependent cellular phagocytosis (ADCP), or macrophage activation are actively explored to amplify antitumor efficacy [358–360].

CD47 is an extensively studied “don’t eat me” signal for phagocytic cells. It can be recognized by signal regulatory protein alpha (SIRPα, an ITIM-bearing inhibitory receptor expressed on macrophages and DCs) to suppress phagocytosis [19]. Importantly, CD47 inhibition utilizing specific antibody was shown to increase phagocytosis-capable TAMs, particularly when used in combination with chemotherapeutic drugs [361]. Additionally, macrophage trogocytosis also serves as an important biological process intervening on antibody-opsonized tumor cells. Unlike conventional phagocytosis, trogocytosis is a specialized process in which part of the cell membrane of the donor cell is consumed through a “bite”, resulting in the transfer of membrane components between cells [362, 363] or even the death of the donor cell [364]. Studies have verified that CD47 blockade bolsters anti-tumor responses through macrophage trogocytosis in renal cell carcinoma and diffuse large B cell lymphoma [365, 366]. Consequently, CD47 blockade represents a promising target for cancer therapy.

Furthermore, as macrophages form a bridge between innate and adaptive immune systems, the combination of phagocytosis checkpoint inhibitors and the current ICI-based immunotherapies are expected to be highly effective in modulating both innate and adaptive antitumor immune responses [367]. To this end, a bispecific antibody targeting PD-L1 on tumor cells and SIRPα on APCs was shown to produce enhanced cytotoxicity to murine colon cancer cells when compared with either anti-PD-L1 or anti-SIRPα monotherapy alone [368]. Surprisingly, a recent report indicates that Sirpα may also limit macrophage phagocytosis in a CD47-independent manner to enhance immunotherapy efficacy, indicating the existence of novel molecular targets and signaling pathways [369]. Currently, a few anti-CD47 monoclonal antibodies, including Hu5F9-G4, CC-9002, ALX148, and HX009, are under clinical trial evaluation with ICIs.

CD24 is another novel “don’t eat me” protein highly expressed in some tumor types. CD24/Siglec-10 signaling (inhibitory receptor sialic-acid-binding Ig-like lectin 10) subverts the immune surveillance of tumor cells. The blockade of CD24/Siglec-10 signaling was found to promote the macrophage-mediated phagocytosis of CD24-expressing tumors and effectively inhibit tumor growth. Moreover, the combination of anti-CD24 and anti-CD47 monoclonal antibodies was shown to remarkably promote the phagocytosis of cancer cells by macrophages, thus suggesting a plausible synergistic antitumor effect from two classes of phagocytosis checkpoint inhibitors [196, 370].

As the intersection of innate immunity and adaptive immunity, the exploration and application of known phagocytic checkpoint inhibitors, as well as the exploration of new phagocytic checkpoints, will make significant contributions to the development of more favorable tumor treatment options in the future.

#### Nanoparticles in the optimization of macrophage-targeted strategies

Systemic targeting of TAMs with nanomedicines has emerged as a promising approach in cancer therapy because of high specificity and reduced side effect. As TAMs have a natural tendency to take up nanomaterials, it has been reported that the cellular uptake of nanoparticles by TAMs was ten times higher than that by tumor cells [195]. Various pharmaceutical cargoes, including TLR agonists, chemotherapeutic drugs, and drugs targeting the phagocytic signals, have been loaded into nanoparticles for delivery to TAMs. To enhance the targeting capacity of nanoparticles, surface modifications of the nanoparticles using ligands, immunoglobulins, or short peptides have been investigated. The combination of these TAM-targeted nanoparticles with chemotherapy or immunotherapy were shown to selectively promote macrophage elimination [371] or reprograming [372], thereby maximizing the anti-tumor efficacy. In order to attain optimal therapeutic outcomes, several issues and challenges remain to be solved. Nanoparticles should be designed to achieve selective delivery to M2-like TAMs and more potent and durable therapeutic responses. Further studies are warranted to design new nanoparticle formulations for TAM-targeted strategies.

## Conclusions

Recent studies have gradually revealed the regulatory functions of TAMs in tumor progression and therapy responses and explored TAM-targeted treatments. Several TAM-targeting strategies including reducing TAM recruitment to the TME, depleting TAMs, repolarizing TAMs toward M1-like macrophages, and blocking ‘don’t eat me’ signals have been investigated with an aim to improve current cancer immunotherapies. Early clinical trials have focused on reducing the number of TAMs. However, instead of diminishing the infiltration of TAMs, the promotion of TAM differentiation towards the antitumor effectors may be a better alternative and this promising strategy is being actively investigated. However, the remarkable heterogeneity and plasticity of TAMs in diverse tumor types, stages and locations are still limiting clinical applications of this strategy to some extent. In addition, TAMs serve as a bridge between innate immunity and adaptive immunity. As mentioned above, on one hand, macrophages rapidly recognize PAMPs or DAMPs through pattern recognition receptors (e.g., TLRs), initiating innate immune responses such as phagocytosis and the release of inflammatory cytokines (IL-1β TNF-α). On the other hand, they present antigenic peptides via MHC-II molecules and express co-stimulatory molecules (CD80/CD86) to directly activate CD4 + T cells, while secreting cytokines like IL-12 to drive Th1 differentiation, thereby orchestrating adaptive immune responses. This unique “sensor-amplifier” characteristic endows them with therapeutic prospects in immunotherapy. Furthermore, combination therapy is also a popular star. To better eradicate the risks or compensatory effects of TAM-targeted strategies and maximize the efficacy of cancer therapy, TAM-targeted approaches are being combined with chemotherapy, immunotherapy and nanoparticles.

With the development of modern techniques including single-cell analyses, precise molecular and metabolic cross-talks in TME will be unveiled in depth, allowing us to identify the crucial mechanisms regulating anti-tumor immune responses mediated by TAMs and explore novel potential targets to optimize the application of TAM-targeted therapies clinically. The more specific classification and relevant biomarkers of TAMs will help in learning about the heterogeneity of TAMs and contribute to overcoming the current restricting factors of TAM-targeted therapies such as limited individualized treatment strategies, precise delivery, drug resistance and inadequate treatment response. We firmly believe that completely unmasking the detailed molecular events and metabolic reprogramming around TAMs in the TME that affect tumor progression and treatment response could contribute remarkably to efficient, individualized, combined and precision medicine in cancer and shed light on drug resistance and cancer therapies.

## Data Availability

Not applicable.

## Abbreviations

Arg1: Arginase 1
ALDH: Aldehyde dehydrogenase
CAFs: Cancer associated fibroblasts
Chi3L1: Chitinase 3-like 1
CLL: Lymphocytic leukemia
CRC: Colorectal cancer
CSCs: Cancer stem cells
CTLs: Cytotoxic T lymphocytes
EGF: Epidermal growth factor
EMT: Epithelial to mesenchymal transition
FGF: Fibroblast growth factor
GIST: Gastrointestinal stromal tumor
GM-CSF: Granulocyte-macrophage colony-stimulating factor
HCC: Hepatocellular carcinoma
HNSC: Head and neck squamous cancer
HSC: Hematopoietic stem cells
ICD: Immunogenic cell death
ICI: Immune checkpoint inhibitor
IDO: Indoleamine 2,3-dioxygenase
IFNγ: Interferon-γ
IL-10: Interleukin-10
KCs: Kupffer cells
KLF3: Transcriptional repressor Kruppel-like factor 3
LIF: Leukemia inhibitory factor
LPS: Lipopolysaccharide
MCP-1: Monocyte chemotactic protein-1
M-CSF: Macrophage-colony stimulating factor
M-MDSC: Monocytes and mononuclear myeloid suppressor cells
MSC: Mesenchymal stem cell
NK cells: Natural killer cells
PCA: Prostate cancer
PDAC: Pancreatic ductal adenocarcinoma
PDGF: Platelet-derived growth factor
PD1: Programmed Death 1
PD-L1: Programmed death ligand 1
PI3Kγ: Phosphatidylinositol 3-kinase γ
PPARγ: Proliferator receptor gamma
RCC: Renal cell carcinoma
scRNA-seq: Single-cell RNA-sequencing
SDF-1: Stromal cell-derived factor-1
Siglec-10: Sialic acid-binding Ig-like lectin 10
SIRPa: Signal regulatory protein alpha
SLL: Small lymphocytic lymphoma
TAMs: Tumor-associated macrophages
STAT3: Signal transducer and activator of transcription 3
TANs: Tumor-associated neutrophils
TGF-β: Transforming growth factor-β
TGTC: Tenosynovial giant cell tumor
TME: Tumor microenvironment
TNBC: Triple negative breast cancer
TNF-α: Tumor necrosis factor-α
Tregs: Regulatory T cells
TREM-1: Triggering receptor expressed on myeloid cell-1
TSG-6: TNF-stimulated Factor 6
VEGF: Vascular endothelial growth factor
ZA: Zoledronic acid
